# Potential of Diffusion-Weighted Imaging in the Characterization of Malignant, Benign, and Healthy Breast Tissues and Molecular Subtypes of Breast Cancer

**DOI:** 10.3389/fonc.2016.00126

**Published:** 2016-05-23

**Authors:** Uma Sharma, Rani G. Sah, Khushbu Agarwal, Rajinder Parshad, Vurthaluru Seenu, Sandeep R. Mathur, Smriti Hari, Naranamangalam R. Jagannathan

**Affiliations:** ^1^Department of Nuclear Magnetic Resonance, All India Institute of Medical Sciences, New Delhi, India; ^2^Department of Surgical Disciplines, All India Institute of Medical Sciences, New Delhi, India; ^3^Department of Pathology, All India Institute of Medical Sciences, New Delhi, India; ^4^Department of Radiodiagnosis, All India Institute of Medical Sciences, New Delhi, India

**Keywords:** diffusion-weighted MRI, breast cancer, apparent diffusion coefficient, benign lesions, molecular biomarkers, estrogen receptor, progesterone receptor, human epidermal growth factor receptor 2

## Abstract

The role of apparent diffusion coefficient (ADC) in the diagnosis of breast cancer and its association with molecular biomarkers was investigated in 259 patients with breast cancer, 67 with benign pathology, and 54 healthy volunteers using diffusion-weighted imaging (DWI) at 1.5 T. In 59 breast cancer patients, dynamic contrast-enhanced MRI (DCEMRI) was also acquired. Mean ADC of malignant lesions was significantly lower (1.02 ± 0.17 × 10^−3^ mm^2^/s) compared to benign (1.57 ± 0.26 × 10^−3^ mm^2^/s) and healthy (1.78 ± 0.13 × 10^−3^ mm^2^/s) breast tissues. A cutoff ADC value of 1.23 × 10^−3^ mm^2^/s (sensitivity 92.5%; specificity 91.1%; area under the curve 0.96) to differentiate malignant from benign diseases was arrived by receiver operating curve analysis. In 10/59 breast cancer patients, indeterminate DCE curve was seen, while their ADC value was indicative of malignancy, implying the potential of the addition of DWI in increasing the specificity of DCEMRI data. Further, the association of ADC with tumor volume, stage, hormonal receptors [estrogen receptor (ER), progesterone receptor (PR), and human epidermal growth factor (HER2)], and menopausal status was investigated. A significant difference was seen in tumor volume between breast cancer patients of stages IIA and IIIA, IIB and IIIA, and IIB and III (B + C), respectively (*P* < 0.05). Patients with early breast cancer (*n* = 52) had significantly lower ADC and tumor volume than those with locally advanced breast cancer (*n* = 207). No association was found in ADC and tumor volume with the menopausal status. Breast cancers with ER−, PR−, and triple-negative (TN) status showed a significantly larger tumor volume compared to ER+, PR+, and non-triple-negative (nTN) cancers, respectively. Also, TN tumors showed a significantly higher ADC compared to ER+, PR+, and nTN cancers. Patients with ER− and TN cancers were younger than those with ER+ and nTN cancers. The present study demonstrated that ADC may increase the diagnostic specificity of DCEMRI and be useful for treatment management in clinical setting. Additionally, it provides an insight into characterization of molecular types of breast cancer and may serve as an indicator of metabolic reprograming underlying tumor proliferation.

## Introduction

Breast cancer is a heterogeneous tumor exhibiting five molecular types, classified by gene profiling and the expression of hormonal receptors such as estrogen receptor (ER), progesterone receptor (PR), and human epidermal growth factor (HER2) (1). The pre-operative tumor size nodal status metastasis (TNM) stage, tumor grade, receptor status, lymph node status, and Ki-67 were outcome predictors of breast cancer in previous studies (2, 3). Accordingly, early diagnosis of malignancy in individual patients may be important for a successful therapy. Mammography is the primary diagnostic screening tool, despite its limitation in sensitivity and specificity, especially in regard to the dense breast (4, 5). In such cases, ultrasound is useful. However, it has limitations in detecting microcalcification and ductal carcinoma *in situ* (DCIS) (6). Breast magnetic resonance imaging (MRI) has become an important adjunct modality in the evaluation of suspicious mammographically occult breast lesions, detection of tumor recurrence, and screening of women with high-risk cancer and those with breast implants (7). It is also useful in pre-operative tumor staging and in the assessment of post-therapy residual disease in a clinical setting (7). The use of dynamic contrast-enhanced (DCE) MRI with gadolinium-based contrast agents depicts high-resolution tumor morphology and allows for contrast uptake kinetics, tumor angiogenesis, and vascularity (8). The DCEMRI has been shown to have high sensitivity (93–99%) but with variable specificity (37–85%) (9).

The rapid proliferation of cancer is associated with reprograming both the anabolic and catabolic pathways, supporting its growth and altering the intracellular and extracellular milieu. Functional MR imaging technique – such as diffusion-weighted imaging (DWI) – is useful to monitor such changes associated with tumor proliferation (10–12). By measuring diffusion constant of water molecules, DWI provides information about the extracellular and intracellular tissue compartments as well as the altered pathologies during cancer growth. The presence of cell membranes, macromolecules, and organelles restrict the motion of water molecules, and therefore decreases the diffusion constant of water compared to free aqueous solution. This is represented as apparent diffusion coefficient (ADC) (13). ADC has been used in differentiating various tissue pathologies, and DWI has in fact been established as an important adjunct technique with many clinical applications (10, 14). Studies have documented a relationship between the cell density and ADC (12, 15). Applications of DWI include characterization of malignant, benign, and healthy breast tissues (12, 16–18), and monitoring of the therapeutic response of breast tumors (19, 20). A correlation of ADC with the histological features and the enhancement ratios using DCEMRI has been reported (21). The growth patterns of cancer and the architectural features of stroma using DWI (21) and the correlation between ADC with the molecular markers of breast cancer have been reported (22–26). The addition of DWI increases the specificity of DCEMRI (27).

The objectives of the present study were (a) to determine a cutoff value of ADC for the differentiation of malignant, benign, and healthy breast tissues in a large cohort of subjects; (b) to evaluate the potential of quantitative DWI in differentiating various histological types of malignant and benign lesions; (c) to evaluate the potential of ADC in indeterminate DCEMRI findings in a sub-group of patients; and (d) to examine the association of ADC, stage, tumor volume, age, menopausal status, and hormonal receptors in these patients.

## Patients and Methods

### Patients

In this prospective study, a total of 388 subjects, including 259 with breast malignancy, 67 with benign breast pathology attending the breast cancer clinic of our Institute, and 54 healthy volunteers, were recruited during the period of 2007–2015. However, data of 8 subjects [malignant (*n* = 4), benign (*n* = 2), and healthy volunteers (*n* = 2)] were excluded from analyses because of motion and other artifacts. The demographic and histological details were presented in Table 1. American Joint Committee on Cancer (AJCC) TNM criteria were used for clinical staging of patients, which included stage IIA, IIB, IIIA, and IIIB + C. In breast cancer patients, MR was performed prior to therapy with at least 1 week after the core biopsy. The purpose and the methodology of the study were fully explained to all the subjects. All studies were carried out as per the standard regulatory guidelines of the institute ethics committee, which approved the study, and written informed consent was obtained from each subject. Clinical history and physical examination was taken for all patients. An ultrasonography, mammogram [Breast imaging Reporting and Data System (BIRADS) IV and V lesions], and histology (fine needle aspiration cytology/core biopsy) were performed. All patients had clinically palpable lumps, and the size of tumor was measured in two dimensions using a Vernier caliper after palpation. In this study, there were no patients with non-subcutaneous tumors.

**Table 1:** **Demographic details of subjects**.

**Breast cancer (*n* = 259)**
Age in years [mean ± SD (range)]	45.4 ± 10.5 (19–70)
*^a^Histology type*	
	**Number**
Invasive ductal carcinoma (IDC)	182
IDC with ductal carcinoma *in situ* (DCIS)	8
IDC + mucinous carcinoma	6
DCIS + cribriform type	2
Papillary carcinoma	1
Ductal adenocarcinoma	2
Lobular carcinoma	3
Medullary carcinoma	1
Neuroendocrine tumor	1
Malignant phyllodes	1
Pagets disease	1
Fibrous stroma	1
*AJCC stage*	
IIA	52
IIB	49
IIIA	45
III (B + C)	113
*Hormone receptor status*	
ER+	93
ER−	92
PR+	82
PR−	100
HER2+	56
HER2−	84
HER2 2+	23
Triple negative (TN)	26
Non-triple negative (nTN)	155
Triple positive (TP)	13
**Benign lesions (*n* = 67)**	
Age in years [mean ± SD (range)]	30 ± 9.4 (13–61)
*Histology type*	
	**Number**
Fibroadenoma	33
Phyllodes	8
Benign ductal epithelial cells	8
Fibrocystic fibroadenoma	8
Cysts	6
Benign proliferative breast disease	1
Fibroepithelial lesion	1
Sclerosing adenosis	1
Mastitis	1
**Healthy volunteers (*n* = 54)**	
Age in years [mean ± SD (range)]	30 ± 9.4 (13–61)

*^a^Histopathology available for 209 breast cancer patients only*.

Biopsied tissues were subjected to histology and immunohistochemical examinations to determine the expression of hormonal receptors, such as ER, PR, and HER2, status according to the standard published guidelines by the American Society of Clinical Oncology/College of American Pathologists (28). Patients with HER2 expression scores of 0 and 1+ were categorized as HER2 negative (HER2−) and those with the scores of 3+ were categorized as HER2 positive (HER2+). Patients with a score of 2+ were excluded from the analysis since their data of fluorescence *in situ* hybridization were not available. Accordingly, the ER status was available for 185 patients, PR status for 182, and HER2 status for 144 patients, and other details were presented in Table 1.

All patients underwent metastatic workup as per the standard guidelines for clinical staging of the tumor prior to any interventional procedure such as neoadjuvant chemotherapy (NACT) or surgery. Further, the metastatic workup included liver function tests, chest roentgenogram, and ultrasound evaluation of abdomen, pelvis, and bone scan. Patients with metastasis, atypia, claustrophobic, on prior treatment, radial scar, pregnant, using contraceptive pills, metallic implants, pacemaker, etc., and also those not willing to take part in the study, were excluded.

### MR Imaging

MR imaging was performed using a four-channel-phased array breast matrix receiver coil at 1.5 T (Magnetom AVANTO, Siemens Healthcare Sector, Germany). Subjects were positioned with head first in prone position with each breast fitting into the cup of the coil. Following the scout images, short tau inversion recovery coronal images were acquired with repetition time (TR) and time to echo (TE) of 6940 and 58 ms, respectively; slice thickness of 3 mm; and a matrix size of 320 × 256. Also, fat-suppressed MR images were acquired in transverse and sagittal planes (TR and TE of 6270 and 102 ms, respectively; slice thickness = 3 mm with no gap; matrix size = 512 × 440). DCEMRI in the axial plane was carried out for 59 breast cancer patients using a fat-saturated 3D FLASH (fast low angle shot) sequence with the following parameters: TR and TE of 5.46 and 2.53 ms, respectively; flip angle = 12°; matrix size = 305 × 448; and slice thickness = 1.4 mm with no gap. Gadolinium-diethylene triamine pentaacetic acid (Gd-DTPA) contrast agent (0.1 mmol/kg) was injected using automatic injector at a rate of 2 ml/s followed by saline flush. One pre- followed by five post-gadolinium image series were acquired with a total acquisition time of 5.5 min (6 × 55 s).

Diffusion-weighted imaging sequence calibration was carried out using a single compartment phantom for water and acetone prepared in separate containers. The mean ADC for water and acetone were 2.25 ± 0.03 × 10^−3^ and 4.1 ± 0.17 × 10^−3^ mm^2^/s, respectively, which were in agreement with the literature (12). Reproducibility of ADC measurements were checked with repeated measurements, and the coefficient of variance (COV) were within 1 and 4% of error limit for water and acetone, respectively. DW images were acquired in the transverse plane covering both the breasts using a single shot echo-planar imaging sequence with the diffusion gradients applied along the orthogonal direction concurrently to reduce motion artifacts (10). The parameters used for DWI were *b* = 0, 500, and 1000 s/mm^2^; TR = 5000 ms; TE = 87 ms; FOV = 250–350 mm; NSA = 1; EPI factor = 128; acquisition matrix = 128 × 128; and slice thickness = 4–5 mm without any inter slice gap. The total acquisition time was 42 s. A minimum of two *b* values are necessary for the measurement of ADC; however, the curve fitting with more than two *b* values reduces the error in ADC estimation by linear regression method; hence, DWI scan with three *b* values was acquired.

### ADC Measurement

Mean ADC values were calculated using an ADC map and by drawing contiguous circular ROIs of five pixels (size = 0.49 cm^2^) on the lesion (malignant and benign lesions) from each patient and from the entire healthy breast tissue in volunteers. For ADC calculation, the mean number of ROIs used for malignant cases was 20 (range 2–137), 19 (range 2–75) for benign cases, and 36 (range 8–163) for healthy volunteers.

### Tumor Volume Measurement

The tumor volume was measured from MR images by a perimeter method using the formula: volume = ST (*A*1 + *A*2, …, *An*), where ST is the slice thickness and *A* is the area of the tumor (20). The subtracted axial dynamic images were used for volume calculation, while sagittal (T2 fat-saturated) images were used in patients for whom DCEMRI could not be carried out. All slices (with no inter slice gap) in which the tumor was seen were used for volume calculation using free hand ROIs. In six malignant lesions of infiltrating ductal carcinoma (IDC) type, ROIs were drawn twice to find out intra-individual variation, which was later verified by another co-author (Uma Sharma). The difference between the COV of the two measurements (inter-individual) was 0.001, and 95% confidence intervals of the difference (CI) was −1.633 to −1.341, indicating that there was no significant variation between the two measurements. The inter-observer agreement was assessed using intraclass correlation coefficient (ICC). The ICC was 0.99, indicating better reproducibility of volume measurements by two different observers.

### Statistical Analysis

One-way analysis of variance (ANOVA) followed by Bonferroni *post hoc* correction was used for comparisons of mean ADC among malignant, benign, and healthy breast tissues. Further, comparison of mean ADC values was also carried out using the analysis of covariance (ANCOVA), considering age as a covariate since mean age was significantly different among the three groups. Receiver operating curve (ROC) analysis was used to obtain cutoff values of mean ADC for the differentiation of malignant, benign, and healthy breast tissues. The sensitivity and specificity of ADC were also calculated. Student’s *t*-test was used to compare the age (years), mean ADC, and tumor volume between patients with positive and negative hormonal receptor status. ANOVA with Bonferroni *post hoc* correction was used for comparisons of mean ADC and tumor volume among various tumor stages. A *P*-value of ≤0.05 was considered significant. All statistical analyses were carried out using statistical software SPSS 19.0.

## Results

**Figure 1:**
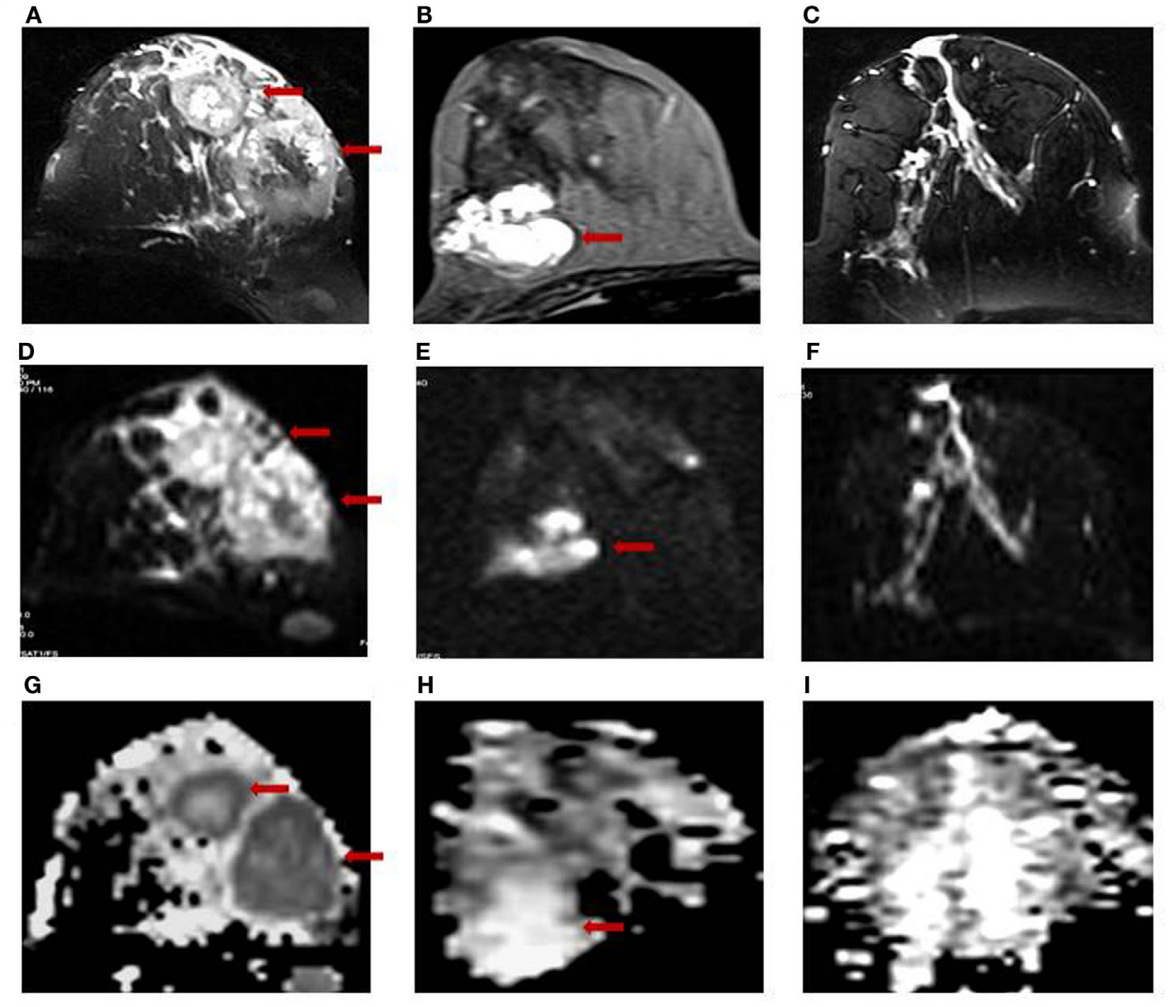
T2-weighted MR images of **(A)** a 28-year-old patient with infiltrating ductal carcinoma (IDC); **(B)** a 25-year-old patient with benign fibroadenoma; and **(C)** a 30-year-old volunteer with healthy breast tissue. The respective DW images are shown in **(D–F)**, while the ADC maps obtained are shown in **(G–I)**.

**Figure 2:**
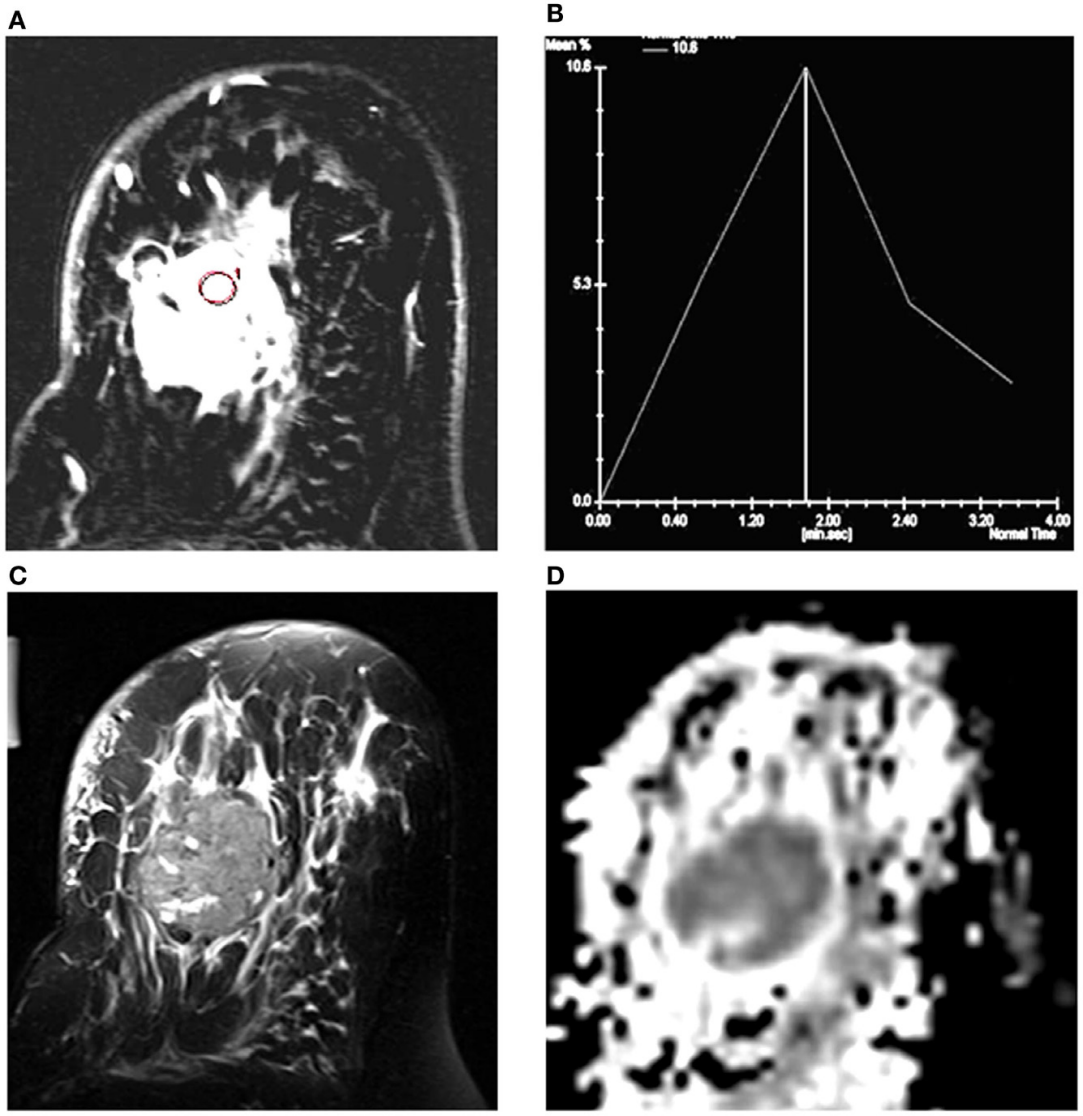
**(A)** T1-axial DCEMR image of a 56-year-old patient with infiltrating ductal carcinoma (IDC); **(B)** Type III dynamic curve with washout pattern from the ROI shown in the lesion; **(C)** T2-weighted fat-saturated axial image; and **(D)** the ADC map of the same patient.

Figure 1 shows the T2-weighted MR image of (A) a 28-year-old patient with IDC; (B) a 25-year-old patient with benign fibroadenoma; and (C) that from a 30-year-old normal healthy volunteer. The DWI images obtained for malignant, benign, and healthy breast tissues are shown in Figures 1D–F, while the respective ADC maps obtained are shown in Figures 1G–I. Figure 2A shows the representative DCE image of a 56-year-old locally advanced breast cancer (LABC) patient with IDC, and the corresponding Type III curve obtained from the ROI positioned in the lesion is shown in Figure 2B. T2-weighted image and the ADC map of the same patient are shown for comparison in Figures 2C,D. The malignant lesion was hypointense compared to the surrounding tissue on T2-weighted image, while on DCE image, the lesion was hyperintense. The time intensity curve showed a washout pattern, which was indicative of malignancy. In addition, on ADC map, the lesion was hypointense, suggestive of restricted diffusion.

### Differentiation of Malignant, Benign Lesions, and Healthy Breast Tissues

The mean ADC of malignant lesions was significantly lower (1.02 ± 0.17 × 10^−3^ mm^2^/s) compared to benign (1.57 ± 0.26 × 10^−3^ mm^2^/s) and healthy (1.78 ± 0.13 × 10^−3^ mm^2^/s) breast tissues (Table 2; Figure 1). Also, a significant difference in the age-adjusted ADC of malignant compared to benign and healthy breast tissues of volunteers was obtained (Table 2). The ADC of various histological types of malignant lesions was similar as shown in Table 3. A comparison of ADC among various benign lesions showed that the ADC was significantly lower in fibroadenomas compared to fibrocystic with fibroadenoma and cystic lesions (*P* < 0.05; Table 4). Further, the ADC of benign ductal epithelial was also significantly lower compared to fibrocystic with fibroadenoma and cystic lesions (*P* < 0.05; Table 4).

**Table 2:** **Distribution of mean ADC in malignant and benign breast lesions, and healthy breast tissue of volunteers and cutoff ADC values using ROC analysis**.

	**Malignant (*n* = 259)**	**Benign (*n* = 67)**	**Healthy volunteers (*n* = 54)**
ADC (×10^−3^ mm^2^/s) (mean ± SD)	1.02 ± 0.17	1.57 ± 0.26*	1.78 ± 0.13*^#^
**ANCOVA**			
ADC (×10^−3^ mm^2^/s)	1.06 (1.04, 1.09)	1.61 (1.55, 1.68)*	1.83 (1.76, 1.89)*^#^
Adjusted mean (95% CI)^@^			
	Malignant vs. benign	Malignant vs. healthy volunteers	Benign vs. healthy volunteers
Difference between means (95% CI)	0.55 (0.55, 0.56)	0.76 (0.76, 0.77)	0.21 (0.21, 0.22)
**ROC analysis and ADC cutoff**			
	Malignant vs. benign	1.23 (sensitivity 92.5%; specificity 91.1%; AUC 0.96)	
	Malignant vs. healthy	1.43 (sensitivity 100%; specificity 98.1%; AUC 0.99)	
	Healthy vs. benign	1.69 (sensitivity 75.9%; specificity 74.6%; AUC 0.79)	

*^@^Adjusted for age*.

*Denotes P < 0.05 between benign vs. malignant; malignant vs. healthy volunteers.

*^#^Denotes P < 0.05 between benign vs. healthy volunteers*.

**Table 3:** **Distribution of mean ADC, volume, and age in various histological types of breast cancer patients (*n* = 209)**.

**Malignant lesions (histological types)**	**Number (*n*)**	**Age (years) (mean ± SD)**	**ADC (×10^−3^ mm^2^/s) (mean ± SD)**	**Volume (cm^3^) (mean ± SD)**
IDC	182	45.4 ± 10.2	1.00 ± 0.16	78.5 ± 89.7
IDC + DCIS	8	43.6 ± 12.9	1.05 ± 0.15	41.3 ± 25.9
IDC + mucinous (or colloid)	6	42.2 ± 19.2	1.05 ± 0.17	59.3 ± 64.0
Papillary	1	53	1.01	29.0
Lobular	3	53.6 ± 7.5	0.96 ± 0.09	73.4 ± 76.7
Ductal adenocarcinoma	2	51.5 ± 9.9	0.91 ± 0.19	42.9 ± 28.2
DCIS + cribriform	2	35.4 ± 14.4	0.97	31.9 ± 19.6
Neuroendocrine	1	65	0.99	15.5
Fibrous stroma	1	53	0.94	29.1
Malignant phyllodes	1	50	1.02	23.5
Paget disease	1	40	0.99	7.2
Medullary	1	57	1.08	30.6
Total	209			

**Table 4:** **Distribution of mean ADC values and age in various histological types of benign breast lesions**.

**Benign lesions**	**Number (*n*)**	**Age (mean ± SD)**	**ADC (×10^−3^ mm^2^/s) (mean ± SD)**
Fibroadenomas (FA)	33	29.1 ± 10.1	1.48 ± 0.17*
Benign ductal epithelial	8	30.9 ± 7.5	1.42 ± 0.18^@^
Benign phyllodes	8	31.3 ± 12.7	1.73 ± 0.25
Fibrocystic with FA	8	29.8 ± 4.4	1.80 ± 0.31*^,^^@^
Cyst	6	33.5 ± 11.5	1.80 ± 0.30*^,^^@^
Sclerosing adenosis	1	29	1.18
Mastitis	1	34	1.44
Fibroepithelial	1	32	1.66
Benign proliferative	1	20	1.79
Total	67		

*P < 0.05 between fibroadenoma and fibrocystic disease with FA and cyst.

*^@^P < 0.05 between benign ductal epithelial cells and fibrocystic disease with FA and cyst*.

Using ROC analysis, a cutoff value of 1.23 × 10^−3^ mm^2^/s [sensitivity of 92.5%; specificity of 91.1% and area under the curve (AUC) of 0.96] was determined to differentiate malignant from benign breast tissues (Table 2).

Furthermore, utility of ADC as an aid in the diagnosis of malignancy in patients with indeterminate DCE findings was evaluated in 59 patients with malignant lesions. Of these, 49 lesions showed a Type III washout curve indicating malignancy, while 9 showed Type II curve, and 1 patient showed Type I curve, suggestive of indeterminate DCE findings (Table 5). However, their ADC values were below the cutoff value (1.23 × 10^−3^ mm^2^/s). Three patients with IDC demonstrated an ADC that was above the cutoff value with a Type III curve on DCEMRI, indicating the false-negative finding (Table 5).

**Table 5:** **ADC value, histology, stage, volume, and BIRADS of breast cancer patients who showed Type I or II curve on DCEMRI and those patients who showed ADC above the cutoff value**.

**Patient no**.	**Histology**	**Curve type**	**ADC (×10^−3^ mm^2^/s)**	**Tumor volume (cm^3^)**	**AJCC stage**	**BIRADS**
1.	IDC	Type I	1.09	77.92	IIB	5
2.	IDC	Type II	1.16	117.6	IIB	5
3.	DCIS + cribriform	Type II	0.97	45.85	IIB	5
4.	IDC	Type II	0.95	2.38	IIIA	4b
5.	IDC	Type II	0.98	78.34	IIB	4
6.	IDC	Type II	1.08	5.26	IIIA	6
7.	Papillary carcinoma	Type II	1.02	29.06	IIB	5
8.	IDC	Type II	0.98	59.82	IIA	3
9.	IDC	Type II	0.97	29.35	IIIB + C	4
10.	IDC	Type II	0.92	44.92	IIIA	4
Breast cancer patients with Type III curve but mean ADC above cutoff						
1.	IDC	Type III	1.31	54.52	IIIA	5
2.	IDC	Type III	1.24	41.43	IIIA	5
3.	IDC	Type III	1.28	160.27	IIIA	5

### Comparison of ADC, Volume, and Age in Malignant Tumors Classified Based on the Stage, Menopausal, and Hormonal Receptor Status

Significant difference in tumor volume was seen, while there was no significant difference in the mean ADC of tumors with different AJCC stages (Table 6). Also, ADC and the tumor volume were significantly higher in LABC patients compared to those with early breast cancer patients (EBC; *P* < 0.05; Table 6).

**Table 6:** **Distribution of mean ADC, age, and volume in various subgroups of breast cancer patients based on tumor stage, menopausal, and hormonal biomarker status**.

**Groups**	**Number (*n*)**	**Age (years) (mean ± SD)**	**ADC (×10^−3^ mm^2^/s) (mean ± SD)**	**Volume (cm^3^) (mean ± SD)**
Premenopausal (Pre)	119	36.4 ± 5.9^§^	1.02 ± 0.18	78.50 ± 80.60
Postmenopausal (Post)	140	53.0 ± 7.5^§^	1.02 ± 0.17	70.45 ± 87.33
EBC	52	46.7 ± 11.9	0.98 ± 0.18^@^	17.67 ± 19.12^$^
LABC	207	45.0 ± 10.4	1.03 ± 0.17^@^	88.17 ± 88.26^$^
Stage IIA	52	46.7 ± 11.9	0.98 ± 0.18	17.67 ± 19.12*
Stage IIB	49	43.8 ± 10.4	0.98 ± 0.12	49.34 ± 51.74*
Stage IIIA	45	46.0 ± 9.2	1.05 ± 0.20	82.23 ± 70.04*
Stage III (B + C)	113	45.2 ± 10.9	1.04 ± 0.17	107.21 ± 101.02*
HER2+	56	44.7 ± 10.3	1.03 ± 0.16	94.60 ± 93.27
HER2−	84	45.1 ± 11.1	1.02 ± 0.15	84.47 ± 83.74
ER+	93	47.8 ± 10.8^£^	0.99 ± 0.14	54.57 ± 50.62^&^
ER−	92	43.4 ± 10.5^£^	1.02 ± 0.16	97.72 ± 104.94^&^
PR+	82	46.9 ± 11.4	1.00 ± 0.14	64.81 ± 61.74^†^
PR−	100	44.4 ± 10.2	1.02 ± 0.16	86.71 ± 100.29^†^
Triple-negative (TN)	26	40.9 ± 9.9^+^	1.07 ± 0.19^#^	111.21 ± 116.34^¥^
Triple-positive (TP)	13	42.8 ± 12.2	1.01 ± 0.16	61.19 ± 69.51^¥^
Non-triple-negative (nTN)	155	46.4 ± 10.8^+^	1.00 ± 0.14^#^	69.21 ± 74.06^¥^

*^§,£,+^Age *P* < 0.05: between ^§^pre vs. post; ^£^ER+ vs. ER−; ^+^TN vs. nTN*.

*^@,#^ADC *P* < 0.05: between ^@^EBC vs. LABC; ^#^TN vs. nTN*.

^$,*,&,†,¥^Volume *P* < 0.05: between ^$^EBC vs. LABC; *stages IIA, IIB, IIIA, and III (B + C); ^&^ER+ vs. ER−; †PR+ vs. PR−; ^¥^TN vs. nTN and TN vs. TP.

*Significant difference in tumor volume of different tumor stages.

Our data further showed that patients who were negative for all receptors, i.e., patients with triple negative (TN) status were of younger age, had a larger tumor volume and a higher ADC value compared to those with non-triple negative (nTN), ER+, and PR+ status. Also, ER− patients showed a larger tumor volume and were of younger age group compared to those with ER+ status (Table 6). The tumor volume of 13 triple positive (TP) patients (positive for all three receptors, i.e., ER+, PR+, and HER2+) was significantly lower than the TN patients, while there was no significant difference in patients’ age and mean ADC value between these two groups. No association was seen between ADC, tumor volume, and the menopausal status of patients (see Table 6).

## Discussion

Metabolic reprograming is an important area of research that combines numerous aspects of metabolic adaptation associated with the cancer proliferation. Cancer cells upregulate their metabolism to meet their biosynthetic demands to facilitate the uncontrolled cell replication, leading to changes in the tissue characteristics and the microenvironment, and these could be monitored using DWI. Due to its potential to improve the characterization and diagnosis of breast lesions, DWI is increasingly being included in breast MRI protocols. The present study investigated its potential in the characterization of breast lesions and its association with prognostic factors, such as tumor stage and hormonal receptor status, in a large cohort of subjects. Our data showed that ADC of malignant lesions was significantly lower compared to benign lesions and healthy breast tissues of volunteers. Also, the age-adjusted ADC of healthy breast tissues was significantly higher compared to the benign breast tissues. These findings were in line with previous studies (29–34). Malignant tumors are characterized by high proliferative activity that increases the cell density and consequently restricts the diffusion of water molecules resulting in a lower ADC value (12, 29, 35). A significant relationship has been reported between ADC and the tumor cellularity using histology (11, 18, 36, 37).

We found 12 different histological types of malignant lesions in our study cohort, which showed similar ADC values. Eight patients with IDC had components of DCIS, and the mean ADC of these lesions was similar to that of IDC lesions. This might be due to increased endoductal cellular density. Several studies have reported higher ADC in DCIS compared to IDC lesions (16, 17, 24, 25, 31, 38). Elucidation of DCIS tumors using DWI has not been consistent due to interspersed distribution of cancer cells and healthy breast parenchyma (17). Medullary invasive cancer, a rare low grade tumor, also showed a low ADC similar to IDC lesions (16, 18, 31). Further, six patients presented with mucinous carcinoma with IDC, and the mean ADC was also similar to that seen for IDC lesions. Few studies have reported a higher ADC for mucinous carcinoma compared to IDC, which has been ascribed to the presence of colloidal mucin content (18) and the relative volume of the mucin and the cellularity of lesion (17, 35). In our study, patients with invasive lobular carcinoma (*n* = 3), Paget’s disease (*n* = 1), fibrous stroma (*n* = 1), and phyllodes (*n* = 1) also showed a lower ADC, similar to that seen for IDC lesions. However, the number of patients in these histological types was less to draw any definitive conclusion.

Additionally, the ADC values were compared among the various histological types of benign lesions. Mean ADC was statistically lower in fibroadenomas and benign ductal epithelial lesions compared to fibrocystic with fibroadenoma and cystic lesions (16, 29). Cystic lesions represent a pouch filled with fluid and proteins, and therefore have less restricted water diffusion compared to that seen in fibroadenomas, which are solid lesions with relatively more cellularity. In literature, lower ADC in fibroadenoma lesions has been reported, which was attributed to the presence of fibrous component (16, 39, 40), while in our study, only one fibroadenoma and one fibrocystic with fibroadenoma lesion showed a low ADC value. Benign phyllodes, mastitis, and benign proliferative lesions showed higher ADC characteristic of benign breast disease. In contrary, a low ADC value similar to malignant tumors has been reported for mastitis (17).

With the availability of ADC values from a large cohort of malignant, benign, and healthy breast tissues, we determined a cutoff value of mean ADC using ROC curves to predict malignancy. A cutoff value of 1.23 × 10^−3^ mm^2^/s was obtained to differentiate malignant from benign disease, which was similar to that reported previously (20, 30, 31, 41). However, a few studies have reported a higher cutoff, which may be due to the variability in experimental parameters used such as low *b* values (14, 16). Further, a meta-analysis of 13 studies that included 615 malignant and 349 benign lesions reported a sensitivity of 84% and a specificity of 79% with area under the curve (AUC) of 0.90 for ADC to differentiate between benign and malignant lesions. The sensitivity and the specificity calculated in this meta-analysis were lower than that seen in the present study (32). This was attributed to the variation in methodology and the *b* values used across studies (32). At low *b* values (<200 s/mm^2^), the effect of perfusion is more, while at high *b* values, pure diffusion component would dominate the measured signal (42). The *b* values used by us emphasize on minimizing the perfusion and T2 shine-through effect. Also, a cutoff value of 1.43 × 10^−3^ mm^2^/s (sensitivity 100%; specificity 98.1%) was obtained to discriminate malignant from healthy breast tissue, which was similar to our earlier observation (20).

Further, we compared the DCEMRI kinetics and the ADC data in detecting malignancy in 59 breast cancer patients. Of the 59 lesions in these patients, 49 showed washout curve (Type III), which was characteristic of malignancy, while 10 showed indeterminate curves [Type I (*n* = 1) and Type II (*n* = 9)]. However, the ADC values obtained in these 10 lesions were below the cutoff value (1.23 × 10^−3^ mm^2^/s), indicative of positive for malignancy. Of these 10 lesions, 8 were IDC, 1 was DCIS + cribriform, and the other 1 was papilloma. Kuhl et al. also have reported DCE kinetics to be inconsistent for diagnosis of DCIS lesions (8). Thus, our findings demonstrated that addition of DWI increases the sensitivity and the diagnostic accuracy of breast cancer (17, 26, 30, 43). Also, DWI has a significant advantage over DCEMRI as diffusion is highly sensitive to changes in the cellular environment, and there is no need to use contrast agent (17). Additionally, DWI has a short scan time. It has also been reported that DWI of breast in combination with T2-weighted imaging has the potential to improve the specificity of breast cancer diagnosis (17). However in our study, three IDC patients with AJCC stage III A and with Type III curve showed an ADC value which was above the cutoff, which may be attributed to the intermingling necrotic cores seen in such large sized tumors (44).

Further, the association among ADC, age, hormonal receptor status, tumor volume, stage, and the menopausal status of patients was investigated. Tumor volume and ADC showed no association with the menopausal status. The tumor volume was significantly different among various AJCC stage tumors, while ADC was not significantly different. LABC patients had a higher ADC and a larger tumor volume compared to EBC patients. Correlation between ADC and the histologically determined tumor size has been reported (36). Higher ADC in large sized tumors may be related to the formation of intermingling necrotic regions due to non-uniform supply of nutrients in fast growing tumors. It is well recognized that by upregulating their metabolism, cancer cells eventually grow into a tumor mass, and further growth requires abundance of nutrients to support biosynthesis of nucleic acids, proteins, and lipids essential for replication. However, the nutrient supply for cancer cells varies across the tumor mass, and the cells on the surface of the tumor may get more nutrients and continue replicating, but cells at the central regions may die due to limited availability of nutrients (45, 46). Further, the gradients in nutrient availability in different regions of the tumor are an outcome of altered metabolic pathways such as glucose metabolism, glutamine synthesis, and oxygen availability (47). Thus, there will be lack of uniform availability of nutrient material affecting the metabolic activity and the viability of tumor cells. This eventually would lead to a heterogeneous mixture of dead, quiescent, and mitotic cells in large sized tumors, which is reflected in parameters such as ADC and tCho concentration determined by functional MR techniques. It was documented that ADC reflects the relative necrotic content of tumors as relative volume fraction of water in the extracellular space is increased due to cell death, leading to higher ADC (48). Further, EBC had a higher tCho concentration compared to LABC, indicating more necrotic cores in LABC (49). In an earlier study, ADC was used to delineate necrotic and viable regions using DWI in patients who cannot afford the cost of contrast (44). In the calculation of ADC, the visible hyperintense necrotic areas were avoided; however, there is a possibility that intermingling microsized necrotic regions might have been included in the ROIs, especially in large tumors, which might have led to higher ADC seen in LABC patients.

Triple-negative cancers showed a higher ADC compared to nTN cancers, which is in agreement with the findings of Youk et al. (23). Also, TN cancers had a larger tumor volume compared to nTN cancers due to aggressive and high proliferative activity (23). It has been established that metabolic reprograming of both catabolic and anabolic pathways occurs to support the survival and high proliferation of cancer cells (47). This metabolic demand is fulfilled by the overexpression of several enzymes such as pyruvate kinase and glutamine fructose 6-phosphate transaminase (50). Higher glutamine utilization compared to other breast cancer types has been reported in TN cancers, indicating higher energy demand for cell proliferation (51). Further, lipid reprograming with upregulation of genes involved in lipid biosynthesis has also been reported to be an important hallmark of cancer development and progression (52). However, TN cancers had lower concentration of tCho (membrane metabolites) compared to that seen in nTN and TP cancers (51). This observation indicates the presence of intermingling necrotic cores in large sized TN cancers and the molecular heterogeneity of breast lesions (51). It was reported that 56% of TN cancers showed intratumoral necrosis (23) and frequent rim enhancement, suggestive of high angiogenesis in the periphery of the tumor, central necrosis, and fibrosis (53). Additionally, the TN group consisted of younger and premenopausal women as reported in literature (54, 55).

Further, our results revealed that ER− cancers had a larger volume and were of younger age compared to ER+ cancers (56, 57). Higher proliferative activity (58) and higher micro-vessel density associated with a larger volume (59) has been documented in ER− cancers. Studies have reported lower ADC in ER+ compared to ER− cancers (25, 42, 56, 60), while the value was similar for these cancers in our study. PR+ showed a lower ADC compared to PR− cancers (25, 61), while a higher ADC has been reported in HER2+ cancers (53, 56, 60, 62). In the present study, there was no significant difference in ADC of HER2+ and PR+ compared to HER2− and PR− cancers. Many studies showed no association of ADC with the hormonal receptor status (22, 24, 63, 64). Thus, variable findings are seen in literature on the association of hormonal receptor status with the ADC values in breast cancer patients.

Several factors may be responsible for such variable findings in the ADC values and its association with the hormonal status of breast cancer patients. First, it may represent the heterogeneous nature of breast cancers, which may be related to the biological behavior of cancers in relation to the expression of various receptors. Inhibition of angiogenic pathway due to estrogen hormone has been reported in ER+ cancers, which may decrease the perfusion in ER+ cancers (22, 65). Recently, Cho et al. reported that higher ADC in ER− cancers may be related to the tumor vascularity and perfusion using intravoxel incoherent motion imaging (42). Hyder et al. have shown that progesterone may increase the angiogenesis through regulation of VEGF in breast cancer (66). Association between angiogenesis and HER2 expression has also been described (67, 68). These findings indicated that positive ER expression was associated with the inhibition of angiogenesis, while positive PR and HER2 expression was associated with the enhancement of angiogenesis. Thus, there is a need to take into account the expression status of all the three receptors while interpreting the ADC and the tumor volume data.

Additionally, the variability in results across studies might also be due to the differences in the tumor size and variations in terms of experimental procedures used such as selection of *b*-value, choice of imaging sequence, and the method used for measurement of ADC. In our study, the mean ADC calculation was carried out by drawing small circular ROIs encompassing the entire visible tumor on a slice that showed the largest tumor area but avoiding necrotic areas. However, most studies in the literature have drawn either single or only few ROIs and have reported mean ADC (24, 60, 62). The methodology adopted by us, though time consuming, may provide a more accurate determination of the ADC and tumor heterogeneity. Arponent et al. also demonstrated that ADC measurements using small ROI were more accurate than whole region ROI (61).

In spite of the clinical potential of DWI, the study has some limitations. First, the sample size was small for some histological types to arrive at definitive conclusions and thus warrant further investigation in a large cohort. Second, multicenter studies with standardized DWI protocol across various laboratories with an appropriate algorithm are required for consistent results in ADC measurement. Third, due to poor socioeconomic status, DCEMRI could not be carried out in all patients.

## Conclusion

The present study on a relatively large cohort of subjects demonstrated that a low ADC value is indicative of malignancy. The sensitivity and specificity calculated from the present data indicated that ADC is a sensitive parameter for the differentiation of malignant, benign, and healthy breast tissues in a short scan time. Further, the changes seen in ADC with various hormonal receptors show its dependence on the biological features of different tumor subtypes, stage, etc. Moreover, the functional MR imaging techniques, such as quantitative DWI, provide an insight into metabolic reprograming and the heterogeneity of breast cancers. Also, the MR imaging features of various molecular subtypes of breast cancer may aid in appropriate incorporation of non-invasive approaches for molecular characterization of breast cancer, which would be useful in treatment planning and patient management.

## Author Contributions

NRJ and US conceived the hypothesis and NRJ, US, RP, and VS designed the experiments. RP and VS recruited patients and carried out clinical work-up, while SM carried out histopathological evaluation and SH helped with DCEMRI data. RS and KA performed MR experiments. RS, US, KA, and NRJ analyzed and interpreted the data. US and NRJ wrote the manuscript that was reviewed by all authors and approved.

## Conflict of Interest Statement

The authors declare that the research was conducted in the absence of any commercial or financial relationships that could be construed as a potential conflict of interest. The reviewer RC and handling Editor declared their shared affiliation, and the handling Editor states that the process nevertheless met the standards of a fair and objective review.
